# Effective screening methods to prevent surgical site infections in orthopedic surgery: an observational study

**DOI:** 10.1186/s12891-023-06471-1

**Published:** 2023-05-06

**Authors:** Tatsuki Kobayashi, Tetsuhiro Ishikawa, Joe Katsuragi, Mitsutoshi Ota, Takanori Omae, Yasuhito Sasaki, Yousuke Tsurumi, Takashi Nomoto, Seiji Ohtori

**Affiliations:** 1 Orthopedic Surgery, Sanmu Medical Center, 167 Naruto, 289-1326 Chiba, Sanmu city, Chiba Japan; 2 Department of Orthopaedic Surgery, Graduate School of Medicine, Chiba University, 1-8-1 Inohana, Chuo-ku, 260-8670 Chiba, Japan

**Keywords:** Nasal culture, Nasal bacterial microbiota, Surgical-site infections, Endogenous infection, Decolonization, Mupirocin

## Abstract

**Background:**

The bacterial source of surgical-site infections (SSIs) can have either endogenous and/or exogenous origins, and some studies have revealed that endogenous transmission is an important pathway for SSIs in orthopedic surgery. However, since the frequency of SSIs is low (0.5–4.7%), screening all surgery patients is labor-intensive and cost-prohibitive. The goal of this study was to better understand how to improve the efficacy of nasal culture screening in preventing SSIs.

**Methods:**

Nasal cultures for 1616 operative patients over a 3-year period were evaluated for the presence of nasal bacterial microbiota and the species identity. We also investigated the medical factors that influence colonization and evaluated the ratio of agreement between nasal cultures and SSI-causing bacteria.

**Results:**

In a survey of 1616 surgical cases, 1395 (86%) were normal microbiota (NM), 190 (12%) were MSSA carriers, and 31 (2%) were MRSA carriers. The risk factors for MRSA carriers were significantly higher than the NM group in patients with a history of hospitalization (13 [41.9%], p = 0.015), patients who had been admitted to a nursing facility (4 [12.9%], p = 0.005), and patients who were > 75 years of age (19 [61.3%], p = 0.021). The incidence of SSIs was significantly higher in the MSSA group (17/190 [8.4%]) than the NM group (10/1395 [0.7%], p = 0.00). The incidence of SSIs in the MRSA group (1/31 [3.2%]) tended to be higher than that in the NM group, but there was no statistically significant difference (p = 0.114). The concordance rate between causative bacteria of SSI and species present in nasal cultures was 53% (13/25 cases).

**Conclusions:**

The results of our study suggest screening patients with a history of past hospitalization, a history of admission in a long-term care facility, and older than 75 to reduce SSIs.

**Trial registration:**

This study was approved by the institutional review board of the authors’ affiliated institutions (the ethics committee of Sanmu Medical Center, 2016-02).

## Background

Surgical-site infections (SSIs) are important operative complications with a significant morbidity and economic burden [1–6]. The bacterial source of SSIs can have either endogenous or exogenous origins [7, 8]. Endogenous SSI can be caused by the transmission of bacteria from the nose, skin surfaces, or other body regions to the surgical site; exogenous infections are caused by bacterial cross-contamination from surrounding patients, health-care workers, and medical equipment [8, 9].

Some authors reported that *Staphylococcus aureus* isolates from nasal carriers and patients with SSIs cluster into the same few clonal complexes [7–9]. For instance, Skråmm reported an 85.7% ratio of concordance in the genetic relationships between wound and nasal isolates in patients who carried *S. aureus* and later developed an SSI [7]. Perl et al. reported that 84.6% of the SSI patients that carried *S. aureus* in their nares, were infected by an identical isolate [10]. These reports strongly suggest that endogenous transmission is an important cause of SSIs in orthopedic surgery.

However, since the frequency of SSIs is low (0.5–4.7%), performing nasal cultures on all patients is labor-intensive and cost-prohibitive [11, 12]. Whether nasal culture testing should be targeted to the elderly, by the presence of comorbidities, or a history of antibiotic use, the targets and methods of screening to effectively prevent SSIs have not been established.

Previous reports suggest that decolonizing the anterior nares prevents *S. aureus* infections among patients receiving dialysis, thereby decreasing complications and costs [13–16]. Several retrospective studies have reported lower SSI rates among patients who received mupirocin [17–19].

In this study, we performed nasal colonization testing on all the operative patients in our hospital and investigated the relationship between nasal bacterial microbiota and SSIs as well as the factors that influence SSI incidence.

## Materials and methods

### Culture isolation and identification

From August 2016 to November 2019, we performed nasal swab cultures on all orthopedic department inpatients upon admission. Specimens were collected by a nurse using dry nasal swabs (sterile disposable swabs) and tested by a clinical laboratory technician. There were 2,804 patients who underwent surgery during this time in our hospital. According to the protocol, eradication was performed on all patients undergoing surgery. After excluding patients who did not receive a nasal culture, such as emergency admissions and those with multiple surgeries, a total of 1,616 patients were included in this study. The surgeries included 931 musculoskeletal traumatic surgeries, 219 spinal surgeries, 223 arthroscopic surgeries, 156 arthroplasties, 16 hand surgeries, and 71 other surgical procedures.

### Data collection and analysis

Data collection was performed by viewing electronic medical records. To investigate the presence of nasal bacterial microbiota and their species, the patients were divided into three groups: normal microbiota (NM), methicillin-sensitive *S. aureus* (MSSA), and methicillin-resistant *S. aureus* (MRSA) groups. Based on previous reports [20], the factors that influence MRSA colonization were investigated, such as diabetes mellitus (DM), a history of hospitalization within 3 years, living in a nursing home, and being more than 75 years old. To investigate the incidence of SSI in each group, we evaluated the ratio of agreement between nasal and SSI cultures as well as the relationship of decolonizing the anterior nares with mupirocin calcium hydrate ointment. The occurrence of an SSI was defined based on an attending physician diagnosis and treatment with antimicrobials or debridement surgery, based on a retrospective review of medical records. Decolonization of the anterior nares was performed using mupirocin calcium hydrate ointment. Mupirocin calcium hydrate ointment was applied intranasally 3 times/day for 3 days.

We also evaluated the cost of nasal cultures in these cases.

### Statistical analysis

Between group differences were tested using the chi square test for statistical analysis. The level of significance was set to p < 0.05. Logistic regression was performed for all risk factors.

## Results

**Fig. 1:**
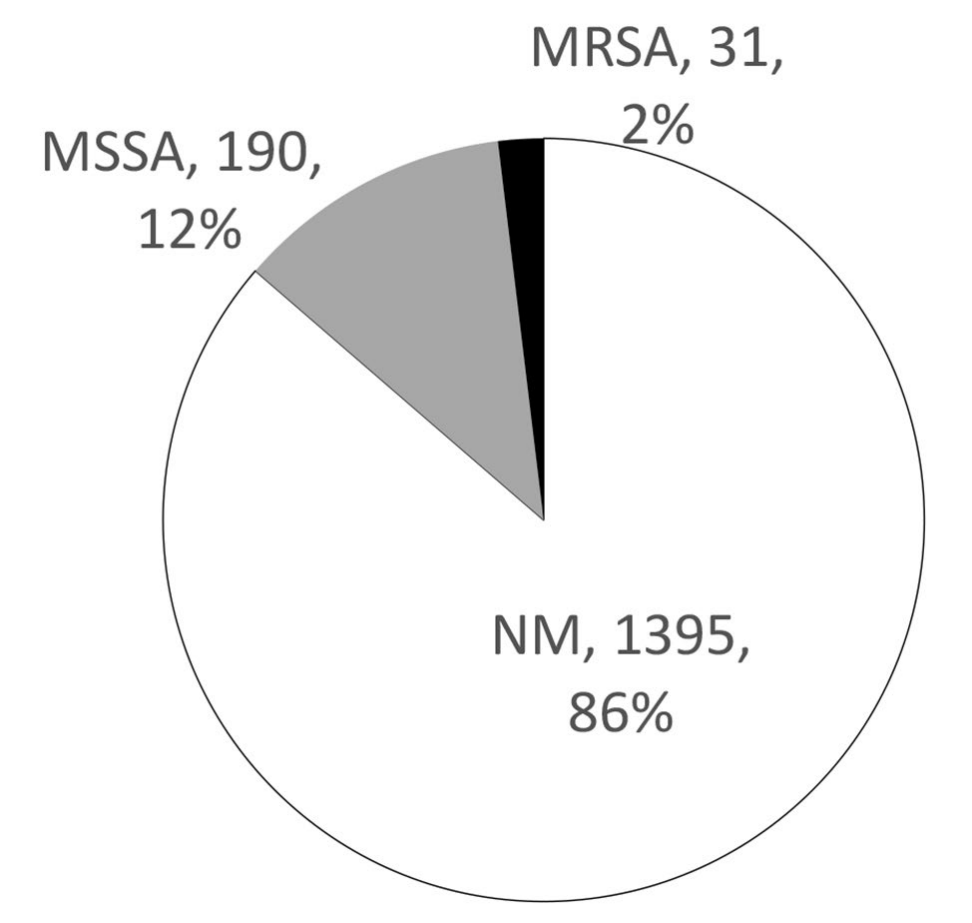
**The result of nasal bacterial microbiota and their species** 1362 out of 1616 (84%) patients had a normal microbiota (NM), 190 (12%) were MSSA carriers, 31 (2%) were MRSA carriers, and 34 (2%) patients had cultures return negative

Of the nasal bacterial cultures, 1,362 out of the 1,616 (84%) were considered normal microbiota (i.e., *S. aureus* was not isolated), 190 cultures (12%) contained MSSA, 31 cultures (2%) contained MRSA, and 33 cultures (2%) were negative for bacteria (Fig. 1). Those who tested negative were analyzed with the NM group. The risk factor analysis of MSSA and MRSA carriers showed that MRSA carriers were significantly more prevalent among patients with a history of hospitalization, living in a nursing home, and more than 75 years old, compared to the NM group. A prior DM diagnosis was not a significant risk factor for MRSA colonization.

With respect to the risk factors for *S. aureus* colonization, the number of patients who had DM was 200/1395 (14.3%) in the NM group, 29/190 (15.2%) in the MSSA group, and 2 /31 (6.5%) in the MRSA group. The number of patients with a history of hospitalization was 323/1395 (23.2%) in the NM group, 32 /190 (16.8%) in the MSSA group, and 13/31 (41.9%) in the MRSA group. The number of patients living in a nursing home was 48/1395 (3.4%) in the NM group, 9 /190 (4.7%) in the MSSA group, and 4/31 (12.9%) in the MRSA group. The number of patients > 75 years of age was 567/1395 (40.6%) in the NM group, 72 /190 (37.9%) in the MSSA group, and 19/31 (61.3%) in the MRSA group. The rates for having a history of hospitalization (p = 0.015), living in a nursing home(p = 0.005), and being older than 75 years (p = 0.021) for patients in the MRSA group are significantly higher than for those in the NM group (Table 1). The results of logistic regression with respect to MRSA nasal swab cultures were as follows: OR = 0.70 for a history of hospitalization within 3 years (95% CI: 0.684–0.706; p < 0.05); OR = 1.50 for living in a nursing home (95% CI: 1.49–1.51; p < 0.05); and OR = 0.47 for being > 75 years of age (95% CI: 0.45–0.49; p < 0.05).

**Table 1:** **Colonization risk factors for MSSA and MRSA.** The rates for having a history of hospitalization, living in a nursing home, and an age above 75 years were significantly higher in the MRSA group than the NM group

	NM n = 1395 (%)	MSSA n = 190 (%)	MRSA n = 31 (%)	p-value (NM vs. MSSA)	p-value (NM vs. MRSA)
Diabetes mellitus	200(14.3)	29(15.2)	2(6.5)	0.733	0.213
Hospitalization *	323(23.2)	32(16.8)	13(41.9)	0.050	0.015
Living in a nursing home*	48(3.4)	9(4.7)	4(12.9)	0.810	0.005
Age older than 75 *	567(40.6)	72(37.9)	19(61.3)	0.468	0.021

*: p < 0.05

**Fig. 2:**
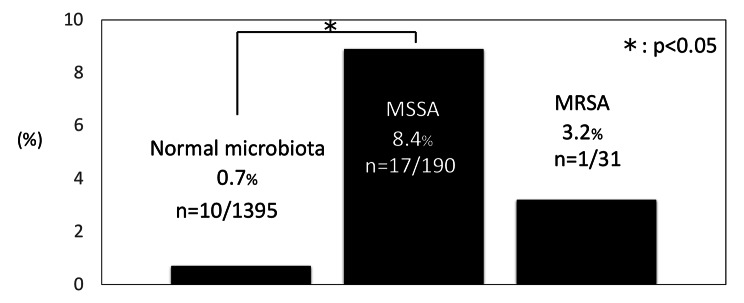
**The incidence of SSI for each group** SSI occurred in 10/1395 (0.7%) patients in the NM group, 17/190 (8.4%) patients in the MSSA group, and 1/31 (3.2%) patients in the MRSA group

SSIs were observed in 28 (1.7%) of the 1,616 patients. The SSI frequency for each group was 10/1395 (0.7%), 17/190 (8.4%), and 1/31 (3.2%) for NM, MSSA, and MRSA, respectively (Fig. 2). The incidence of SSIs was significantly higher in the MSSA group (17/190 [8.4%]) than the NM group (10/1395 [0.7%], p = 0.00). The incidence of SSIs in the MRSA group (1/31 [3.2%]) tended to be higher than that in the NM group, but there was no statistically significant difference (p = 0.114) (Fig. 2). In the MRSA group, MRSA was identified as the causative agent of the SSI. The causative SSI bacteria were identified in 25 of the 28 SSI cases (89%). In 13 cases (53%), the causative bacteria matched the species isolated from the patient’s nasal culture.

Of the 31 MRSA carriers, MRSA was eradicated from 15 patients; six of these patients were later retested and remained MRSA free. In nine of the 31 carriers, the surgery was performed before the test results were available, and therefore, decolonization with mupirocin was not performed before their surgeries.

The cost of one nasal culture test in Japan is $11.60. In this study we tested 1616 patients; therefore, the total nasal culture cost was $18,700. Two cases of MRSA surgical site infections were identified, with an average duration of hospitalization for therapeutic management being three months, incurring an average expense of $25,600.

## Discussion

The rate of MSSA and MRSA carriers in the study was 12% and 2%, respectively. Kawabata et al. reported nasal carrier rates of MSSA and MRSA at 20.1% and 4.4%, respectively in 439 orthopedic patients [21]. The rates of MSSA and MRSA carriers in this study were similar to those reported in previous studies.

The proportion of patients with a history of hospitalization, living in a nursing home, and > 75 years of age in the MRSA group was significantly greater in this study. Wakatake et al. reported that factors correlated with MRSA colonization rates of 30% or more are: a history of past hospitalization (40.0–43.9%), admission to a long-term care facility in the past 5 years (32%), and being at least 75 years of age (33.2%) [20]. In our study, the rates of patients in the MRSA group with a history of hospitalization, living in a nursing home, and an age above 75 years old are significantly higher than those of the NM group. Based on the results of our study and previous reports, nasal cultures may be cost-effective when screening patients who have history of past hospitalization, admission to a long-term care facility, and/or are older than 75 [20].

The incidence of SSI was significantly higher in the MSSA group and the concordance rate between causative bacteria of SSI and the nasal culture species was 53% (13/25 cases) in the current study. The results of these manuscripts revealed that *S. aureus* isolates from nasal carriers and patients with SSI clustered into the same few clonal complexes. We did not perform a genetic analysis and our results were not as consistent as these papers; however, these data support endogenous transmission as an important pathway for SSIs in orthopedic surgery. Although MSSA carriers had the highest SSI rates in this study, it is unclear whether MSSA should be eradicated in carriers [22].

Decolonization of the anterior nares was performed on 15 of the 31 MRSA-carrying patients using mupirocin calcium hydrate ointment; all six patients who were retested after eradication remained MRSA-isolate free. According to Gernaat van et al., in a survey of 2,088 orthopedic surgery cases, the incidence of SSI was 1.3% in the group using nasal MRSA eradication agents and 2.7% in the control group, suggesting that eradication was effective [19]. Because our hospital is an acute care hospital, and surgery is often performed immediately after admission for most trauma cases, we were unable to conduct MRSA-eradication protocols on 16 of the 31 MRSA carriers, and even in the eradicated cases, surgery was performed before the efficacy of eradication could be determined. Furthermore, the incidence of SSI is low in general [11, 12]. Because eradication is effective based on a previous report [19], we need to create a system for *S. aureus* eradication in the nares and improve awareness to promote eradication in future cases.

In this study the total cost of nasal cultures was $18,700. If we only tested patients with 1 of 3 risk factors, 676 of 1616 patients would have been tested, for a total savings of $10,900. In this case 25 of 31 patients (80.6%) with MRSA would be identified and 6 patients (19.4%) with MRSA would be missed. The study revealed an average hospitalization duration of three months for therapeutic management, with an associated cost averaging $25,600. Our investigations demonstrated the SSI incidence rate of 3.2% in the MRSA group. The cost of untested MRSA carriers would amount to $4,920 for six individuals ($25,600 × 6 × 3.2%). Thus, targeted screening measures can be deemed cost-effective.

Our study has several limitations. First, we did not perform genetic analyses, which limited our ability to determine a causal relationship between nasal colonization and SSI isolates. Second, as many of our subjects were emergency trauma admissions, we could not always wait for the culture results before surgery. Thus, about half of our MRSA carriers were not identified and subjected to MRSA-eradication protocols before surgery. Third, this is an observational study in which the diagnosis of SSI was made based on a retrospective examination of medical records.

## Conclusions

We performed nasal colonization testing and investigated the presence and identity of nasal bacterial microbiota for 1,616 patients, all the operative cases in our hospital for three years. The incidence of SSI was significantly higher in MSSA and MRSA carriers, and the concordance rate between causative agents of SSI and the species of nasal culture was 53%. Based on the results of our study, and previous reports, nasal culturing may be cost-effective for screening patients with a history of past hospitalization, a history of admission in a long-term care facility, and who are older than 75.

## Data Availability

The datasets used and/or analysed during the current study are available from the corresponding author on reasonable request.

## List of abbreviations

SSI: surgical-site infections
NM: normal microbiota
MSSA: methicillin-sensitive S. aureus
MRSA: methicillin-resistant S. aureus
DM: diabetes mellitus
